# Supplementary figures and images for: Enhancing semantic segmentation in chest X-ray images through image preprocessing: ps-KDE for pixel-wise substitution by kernel density estimation
Source: PLoS One. 2024 Jun 24;19(6):e0299623. doi: 10.1371/journal.pone.0299623 (PMC11195943; doi:10.1371/journal.pone.0299623)

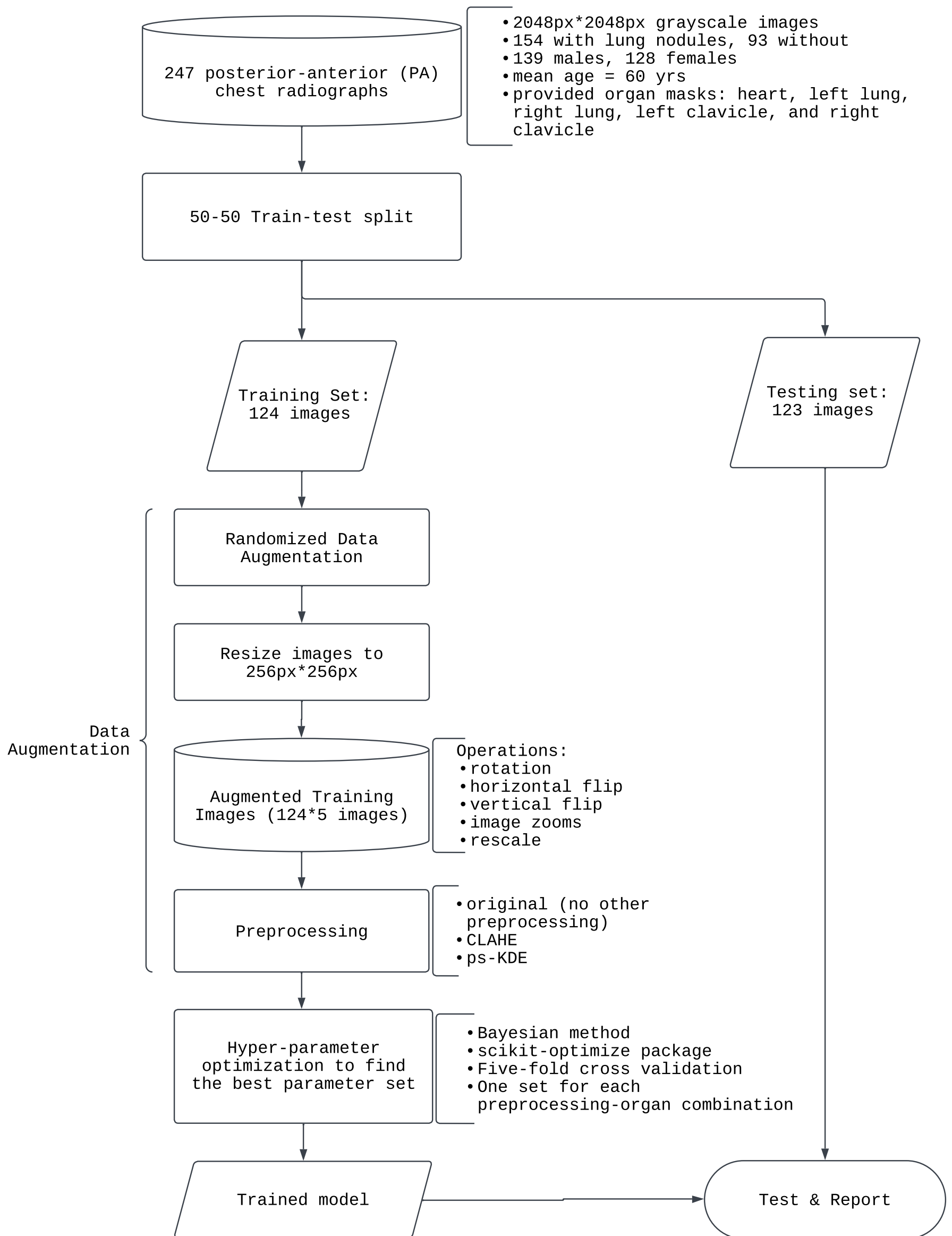

Supplement: S1 Fig — (PDF) [file pone.0299623.s001.pdf]
