# Supplementary material for: Enhancing semantic segmentation in chest X-ray images through image preprocessing: ps-KDE for pixel-wise substitution by kernel density estimation
Source: PLoS One. 2024 Jun 24;19(6):e0299623. doi: 10.1371/journal.pone.0299623 (PMC11195943; doi:10.1371/journal.pone.0299623)
Supplement: Attachment — Submitted filename: PLOS-ONE reviewer-response-Wang.docx [file pone.0299623.s002.docx]

# Authors’ Response to the Reviewers’ Comments

***Journal:*** *PLOS One*

***Title of Manuscript:*** *Enhancing Semantic Segmentation in Chest X-Ray Images through Image Preprocessing: ps-KDE for Pixel-wise Substitution by Kernel Density Estimation*

***Authors:*** *Yuanchen Wang, Yujie Guo, Ziqi Wang, Linzi Yu, Yujie Yan, Zifan Gu*

***Date Sent:*** *04/16/2024*

Dear Editor,

Thank you for your email regarding our revision of the manuscript. We appreciate the comments and suggestions from the reviewers. In the current document, we have addressed each of the issues and questions provided by the reviewers to the best of our knowledge. Corresponding changes have also been applied to our manuscript and highlighted in tracked changes. If more comments are to come, we are happy to further improve our manuscript.

Sincerely,

Zifan Gu

The University of Texas Southwestern Medical Center

Danciger Research Building

5323 Harry Hines Blvd, Ste H9.124, Dallas, TX 75390-8821

zifan.gu@utsouthwestern.edu

**Response for comments from Reviewer 1**

**Comment #1:** The paper should undergo language editing before it can be published.

**Response:**

This manuscript has been edited by a native English speaker with a bachelor's degree.

**Comment #2:** A literature review section is required.

**Response:**

We agree with the reviewer that a literature review section is an important part of the manuscript. Previously, we provided a review of works utilizing CLAHE, a well-established method that serves as a point of comparison, and its impact on deep learning models which included applications in diabetic retinopathy, MRI, and X-rays. In this revision, we have expanded this to include a review of works enhancing contrast through deep-learning approaches, such as a contrast diffusion model and an unsupervised Retinex model. To enhance readability, we have now split the introduction into two sections: background and related works.

**Comment #3:** The evaluation should be for the whole model in terms of loss, accuracy, intersection over union (IoU), dice, sensitivity, specificity, specificity, recall, and precision.

**Response:**

We agree with the reviewer that adding more evaluation metrics could help readers have a more comprehensive understanding of the model performance. Thus, precision, recall, and accuracy have been added in addition to the currently presented IoU and dice scores.

**Comment #4:** The pseudocode should be used to write algorithms 1 and 2. Furthermore, a flowchart will be good if included with algorithms.

**Response:**

The authors agree that a comprehensive description of the algorithm could further improve the readability of the paper. Therefore, we have replaced algorithms with flowcharts in plain language. The executable source code for demonstration is now available on GitHub (<https://github.com/wyc79/ps-KDE>).

**Comment #5:** To improve the readability of the paper, I suggest dividing the analysis into several subsections.

**Response:**

We thank the reviewer for suggesting a clearer organization of the manuscript. To enhance readability, we have introduced several subsections within the 'model development' section, including model architecture, loss function, hyperparameter optimization, and evaluation metrics.

Additionally, we have refined the results section to eliminate redundant reporting of mIOU and F1 scores, which are already presented in Table 3. Instead, we have focused on comparing the two preprocessing techniques using relative languages(e.g. *With CLAHE transformations, the heart model maintained its superior performance, albeit with the right clavicle registering the lowest scores.*).

To further improve readability, we have separated the results sections into “Model Evaluation” and “Model Interpretation”. Within  “Model Evaluation,” we have detailed our reporting to first focus on technique-specific model performances, then a separate paragraph on organ-specific model performances.

**Comment #6:** The authors should explain the dataset split (Training and Testing) rate (90% and 10% or 80% and 20% or 70% and 30%)

**Response:**

We agree with the reviewer that dataset splitting is crucial in studies involving deep learning algorithms. Previously, such information was a part of the model development section, potentially diminishing the manuscript's readability. Consequently, we have addressed this concern by creating a dedicated section in Methods to detail data splitting: “Train/Validation Split”. Specifically, our dataset was divided as follows: *the training set contains 50% of the radiographs (n=124), and the validation contains the other 50% (n=123). Having executed five distinct augmentation techniques individually, we expanded our initial training set to five times its original size, leading to an allocation of 80% for training (n=620) and 20% for validation (n=123).*

**Comment #7:** The author should provide evidence to support that the suggested model is better than the existing models.

**Response:**

We appreciate the reviewer's inquiry regarding the deep-learning (DL) segmentation model utilized in our analysis. It's essential to clarify that our study primarily focuses on assessing the impact of various preprocessing methods on DL model performance rather than comparing different model architectures. The background information and rationale of selecting this specific model is described below and also mentioned in our introduction. According to nn-UNet, a widely recognized segmentation pipeline that ranked the top in the Medical Segmentation Decathlon, preprocessing significantly influences the training performance of deep-learning segmentation algorithms in medical imaging. The model performance is promising and comparable with proper preprocessing and a simple base U-Net. Given this insight, we opted for simpler model architectures at the beginning of our project. Notably, we started with UNet with ResNet backbone, a popular choice, and observed that ResNet34 already yielded satisfactory results.

**Comment #8:** The discussion and conclusions need to be rewritten to highlight the key contributions of this manuscript.

**Response:**

We agree with the reviewer that the discussion and conclusion should reflect the three key contributions we mentioned in the last paragraph of the introduction. Our discussion paragraphs are now structured as 1) the significance of introducing an end-to-end image enhancement method, 2) the efficacy of the data augmentation method in deep learning algorithms, and 3) clinical interpretation made possible by heatmaps.

The conclusion of this manuscript now reads: *In conclusion, we significantly improved semantic segmentation of the left lung in chest radiographs using ps-KDE. ps-KDE is easy to implement, adaptive across diverse datasets, and enhances the robustness of segmentation algorithms. The introduction of the ps-KDE preprocessing technique contributes to the available image contrasting methods for segmentation but should be treated with caution and further validations.*

**Comment #9:** A flowchart for the suggested model is required to show the process steps.

**Response:**

The authors interpret the request for a flowchart for the “suggested model” and “processing steps” as to illustrate how chest radiographs are utilized in the development of ps-KDE and its integration with ResNet34. We agree with the reviewer on the importance of providing a detailed project overview through a flowchart and have incorporated it as Figure S1 in the supplementary figure. For a deeper understanding of the segmentation model architecture, please refer to Figure 5.

**Response for comments from Reviewer 2**

**Comment #1:** Define MRI, CT, and other abbreviation in its first place in the document.

**References:**

Revision completed.

**Comment #2:** It would be good to present both of the algorithms in its standard form, not in image or screenshot.

**Response:**

The authors agree that a comprehensive description of the algorithm could further improve the readability of the paper. Therefore, we have replaced algorithms with flowcharts in plain language. The executable source code for demonstration is now available on GitHub (<https://github.com/wyc79/ps-KDE>).

**Comment #3:** There are advance versions of ResNet like ResNet50 etc , why the authors used ResNet34?

**Response:**

We appreciate the reviewer's inquiry regarding the deep-learning (DL) segmentation model utilized in our analysis. It's essential to clarify that our study primarily focuses on assessing the impact of various preprocessing methods on DL model performance rather than comparing different model architectures. The background information and rationale for selecting this specific model are described below and also mentioned in our introduction. According to nn-UNet, a widely recognized segmentation pipeline that ranked the top in the Medical Segmentation Decathlon (MSD), preprocessing significantly influences the training performance of deep-learning segmentation algorithms in medical imaging. The model performance is promising and comparable with proper preprocessing and a simple base U-Net. Given this insight, we opted for simpler model architectures at the beginning of our project. Notably, we started with UNet with ResNet backbone, a popular choice, and observed that ResNet34 already yielded satisfactory results.

**Comment #4:** Result section is short and poor, please improve.

**Response:**

In order to help readers have a more comprehensive understanding of the model performance results, we have added more evaluation metrics. Specifically, precision, recall, and accuracy have been added in addition to the currently presented IoU and dice scores. Additionally, we have refined the results section to eliminate redundant reporting of mIOU and F1 scores, which are already presented in Table 3. Instead, we have focused on comparing the two preprocessing techniques using relative languages(e.g. *With CLAHE transformations, the heart model maintained its superior performance, albeit with the right clavicle registering the lowest scores.*).

To further improve readability, we have separated the results sections into “Model Evaluation” and “Model Interpretation”. Within  “Model Evaluation,” we have detailed our reporting to first focus on technique-specific model performances, then a separate paragraph on organ-specific model performances.

**Comment #5:** In introduction, add more work as the authors did not include literature as a separate section so the introduction part must be longer.

**Response:**

We agree with the reviewer that a literature review section is an important part of the manuscript. Previously, we provided a review of works utilizing CLAHE, a well-established method that serves as a point of comparison, and its impact on deep learning models which included applications in diabetic retinopathy, MRI, and X-rays. In this revision, we have expanded this to include a review of works enhancing contrast through deep-learning approaches, such as a contrast diffusion model and an unsupervised Retinex model. To enhance readability, we have now split the introduction into two sections: background and related works.

**Response for comments from Reviewer 3**

**Comment #1:** The paper hasn’t mentioned the dataset splitting (train, validation, test), this could give the reader in depth knowledge about the experiment.

**Response:**

We agree with the reviewer that dataset splitting is crucial in studies involving deep learning algorithms. Previously, such information was a part of the model development section, potentially diminishing the manuscript's readability. Consequently, we have addressed this concern by creating a dedicated section in Methods to detail data splitting: “Train/Validation Split”.  Specifically, our dataset was divided as follows: *the training set contains 50% of the radiographs (n=124), and the validation contains the other 50% (n=123). Having executed five distinct augmentation techniques individually, we expanded our initial training set to five times its original size, leading to an allocation of 80% for training (n=620) and 20% for validation (n=123).*

**Comment #2:** The paper sorted the limitation while discussing the results, however, for regions where ps-KDE underperformed (such as the Right Clavicle and Right Lung), I recommend providing a more detailed analysis or hypothesis explaining why this might be the case as well as adding more evaluation metrics which help to give wider look in the performance. Including such a discussion would help in understanding the limitations of ps-KDE and in guiding future improvements.

**Response:**

We agree with the reviewer that adding more evaluation metrics could help readers to have a more comprehensive understanding of the model performance. Thus, precision, recall, and accuracy have been added in addition to the currently presented IoU and dice score. We have then added interpretations of the precision and recall scores, offering hypotheses on why ps-KDE underperformed in certain regions and potential ideas for future improvements. These comments are detailed in the fourth paragraph of the discussion.

**Comment #3:** I also recommend expanding the limitation and discussion to discuss the computational efficiency of ps-KDE compared to other methods, especially if the approach is to be recommended for clinical applications where processing speed can be a critical factor.

**Response:**

We agree with the reviewer that computational efficiency is a critical elements when evaluating whether it can be adopted clinically. Thus, after discussing segmentation performances, we have added a paragraph discussing computational efficiency of CLAHE and ps-KDE. This is now the fifth discussion paragraph starting with “*We would also like to factor in computational efficiency given its potential impact on the integration of algorithms into clinical practice...*”
